# Two-dimensional lamellar polyimide/cardanol-based benzoxazine copper polymer composite coatings with excellent anti-corrosion performance†

**DOI:** 10.1039/d1ra08844k

**Published:** 2022-04-07

**Authors:** Xiangyang Chen, Xinmei Zhang, Jipeng Chen, Weibin Bai, Xiaoxiao Zheng, Qi Lin, Fengcai Lin, Yanlian Xu

**Affiliations:** College of Chemistry and Materials, Fujian Normal University Fuzhou 350007 PR China; Fujian Engineering and Research Center of New Chinese Lacquer Materials, College of Materials and Chemical Engineering, Minjiang University Fuzhou 350108 China linfengcai8@163.com ylxu@mju.edu.cn; College of Materials Science and Engineering, Huaqiao University Xiamen 361021 China; Fujian Key Laboratory of Polymer Materials, Fujian Normal University Fuzhou 350007 PR China

## Abstract

The economic loss and environmental damage caused by metal corrosion is irreversible. Thus, effective methods, such as coating technologies are used to protect metal surfaces from corrosion. In this work, cardanol-based benzoxazine (CB) was synthesized by a solvent-free method using cardanol, paraformaldehyde and *n*-octylamine. A cardanol-based benzoxazine copper polymer (CBCP) with good mechanical properties was then prepared by CuCl_2_ catalysis and can be cured at room temperature. Subsequently, polyimide corrosion inhibitors with a two-dimensional sheet structure (pyromellitic dianhydride polyimide (PDPI) and 1,4,5,8-naphthalene tetracarboxylic dianhydride polyimide (NDPI)) were designed and prepared. Lastly, PDPI or NDPI was mixed with CBCP to obtain two-dimensional lamellar polyimide/cardanol-based benzoxazine copper polymer composite coatings. The Tafel curves and electrochemical impedance spectroscopy (EIS) measurements showed composite coatings with good corrosion resistance in different corrosive media. Compared to CBCP coating, the anticorrosion performance of the composite coatings improved obviously, especially the coating obtained with 0.5 wt% PDPI. It exhibits a high polarization resistance (3.874 × 10^9^ Ω), a high protection efficiency (99.99% and 97.98%) and low corrosion rate (3.376 × 10^−6^ mm year^−1^). This work suggested a facile and eco-friendly strategy for preparing bio-based anticorrosive composite coatings from low cost and abundant cardanol and polyimide corrosion inhibitors, which will significantly promote their application in metal anticorrosion.

## Abstract 2

Room temperature cured two-dimensional lamellar polyimide/cardanol-based benzoxazine copper polymer composite coatings were successfully prepared, which exhibited excellent mechanical properties and anticorrosion properties.

## Graphical abstract

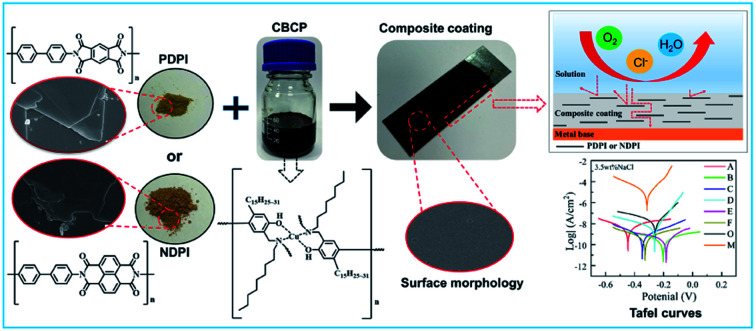

## 1. Introduction

The annual economic losses from metal corrosion amount to $2.5 trillion, accounting for 3% of global GDP.^1^ Hence, metal corrosion requires urgent solutions. Among various corrosion protection strategies, the formation of anticorrosive coatings could efficiently protect metal surfaces at a low cost.^2,3^

Polybenzoxazine resin has become increasingly popular in anti-corrosion composite coatings because of its close to zero shrinkage, low water absorption, high thermal stability, flexible molecular designability, good dielectric characteristics, and superior mechanical properties.^4–6^ For instance, Xin *et al.* synthesized a silane-functional polybenzoxazine coating with good anti-corrosion shielding performance.^7^ The charge transfer resistance (*R*_ct_) of hydrophobic benzoxazine-cured epoxy coating was about 3 fold higher than that of bare steel.^8^ Compared to the traditional petroleum-based materials, the development and utilization of bio-based materials have become gradually well-liked owing to their easy processing and renewable characteristics.^9–11^ Such as cellulose,^12^ cardanol,^13^ alginate,^14^ soybean oil,^15^ and so on. Cardanol, as a natural phenol with abundant yield and easy extraction, was often used in the preparation of anti-corrosion coatings.^16^ Vijayan *et al.* synthesized a colorless, transparent, hydrophobic and corrosion resistant coating with cardanol.^17^ Aggarwal *et al.* developed an epoxy–cardanol resin using cardanol exhibits better properties as compared to epoxy resin in terms of increase in tensile strength, elongation.^18^ There are also many reports about cardanol-based benzoxazines. Patil *et al.* synthesized a bio-based amine functional benzoxazine resin from cardanol shown good mechanical, chemical, and solvent resistance properties.^19^ Huang *et al.* combined cardanol-based polybenzoxazine and amino-modified silica nanoparticles to develop a superhydrophobic coating on mild steel for anti-corrosion coating application.^20^ Hence, cardanol-based benzoxazines with low cost, eco-friendly and excellent mechanical properties are usually prepared from cardanol as a raw material and as a good anti-corrosion matrix material. Nevertheless, the high curing temperature (>200 °C) of benzoxazine limits its application in practical construction process. Therefore, in this work, CuCl_2_ was used to catalyze the ring-opening reaction of cardanol-based benzoxazine and can be cured at room temperature.

On the other hand, the existence of cracks and holes, as well as the inherent permeability of corroded media and degradation of traditional material matrices during curing shrinkage may significantly reduce the barrier efficiencies of coatings.^21^ The two-dimensional lamellar materials, such as graphene and its derivatives, boron nitride and molybdenum disulfide, often used as a corrosion inhibitor in anticorrosive coatings due to their unique lamellar structures and high specific surface areas.^22–24^ Because of special lamellar structure, two-dimensional materials can increase the tortuous diffusion pathways avoiding corrosion mediums directly accessing the substrate surface, leading a good anti-corrosion property.^25^ However, since most common corrosion inhibitors were composed of inorganic materials, the poor dispersion in organic coatings may also decline the shielding performance of coatings.^26^ Polyimide was widely used in the field of aeronautics and astronautics, separation membrane and anticorrosive coatings because of its excellent mechanical energy, electrical insulation, thermal stability and chemical resistance.^27–29^ Therefore, adding two-dimensional lamellar polyimide into resin as a corrosion inhibitor is expected to show significantly effect and further improve the anticorrosion performance of matrix material.

In this work, cardanol-based benzoxazine (CB) was prepared by solvent-free synthesis method using cardanol as raw material. CuCl_2_ was then introduced into catalyze ring-opening polymerization to form cardanol-based benzoxazine copper polymer (CBCP) that can be cured at room temperature yield excellent mechanical properties. Meanwhile, two aromatic polyimides (PDPI and NDPI) with two-dimensional lamellar structures were designed and prepared, and then added into CBCP as corrosion inhibitors. The results show that the synergistic effect induced between two-dimensional lamellar polyimide with highly insulating and CBCP with excellent mechanical properties yielded, two-dimensional lamellar polyimide/cardanol-based benzoxazine copper polymer composite coatings with excellent anti-corrosive properties.

## 2. Materials and methods

### 2.1 Materials

Cardanol was purchased from Jining Hengtai Chemical Co., Ltd, China. Copper chloride (CuCl_2_), *n*-octylamine (99 wt%), xylene, anhydrous ethanol, acetone, dichloromethane (CH_2_Cl_2_), *N*,*N*-dimethylformamide (DMF, ≥99.5 wt%), and anhydrous magnesium sulfate (MgSO_4_, 98 wt%) were all obtained from Sinopharm Chemical Reagent Co. Ltd, Shanghai, China. Paraformaldehyde (95 wt%) and pyromellitic dianhydride (PMDA, 96 wt%) were provided by Shanghai Aladdin Reagent Co., Ltd, China. Benzidine (BZD, 98 wt%) and 1,4,5,8-naphthalene tetracarboxylic dianhydride (NTCDA, 96 wt%) were received from Shanghai Macklin Biochemical Co., Ltd, China. All reagents were used as received without further purification. Deionized water was used throughout the work.

### 2.2 Synthesis of CB and CBCP

**Scheme 1:**
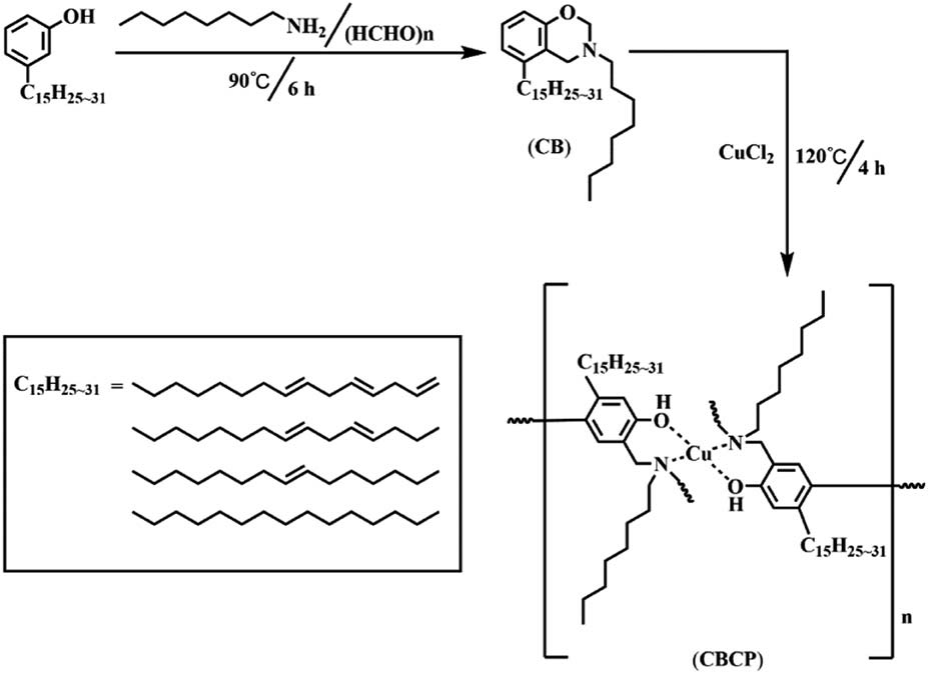
Synthesis route of CBCP.

CB was prepared by solvent-free synthesis. Briefly, 3.05 g cardanol, 0.60 g paraformaldehyde, and 1.29 g *n*-octylamine were added to a round bottom flask and the mixture was left under magnetic stirring and heating at 90 °C for 6 h. Afterward, the system was cooled down to room temperature, and the formed organic layer was extracted with CH_2_Cl_2_ and warm deionized water followed by drying with anhydrous magnesium sulfate for 10 h. The resulting organic solvent was removed by rotary evaporation to yield a brown viscous product, which was dried under vacuum at 40 °C for 24 h. CBCP was synthesized by CuCl_2_ catalyzed ring-opening polymerization. Briefly, 4.58 g CB and 10 mL xylene were first loaded into a round bottom flask, and the mixture was stirred at room temperature for 30 min to yield a homogeneous solution. Next, 0.34 g CuCl_2_ was dissolved in anhydrous 5 mL ethanol and then slowly added to the round bottom flask by a constant pressure drip funnel. After heating to 120 °C for 4 h. CBCP was obtained. The preparation process of CBCP is shown in Scheme 1.

### 2.3 Synthesis of PDPI and NDPI

**Scheme 2:**
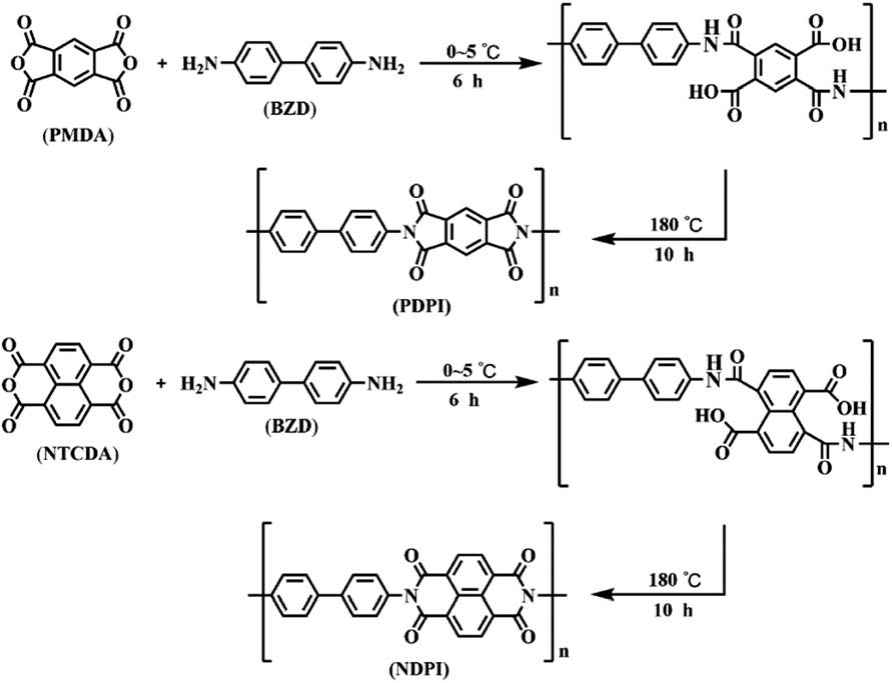
Synthesis route of PDPI and NDPI.

Firstly, 1.84 g benzidine was dissolved in a round bottom flask containing 60 mL DMF. Dianhydride monomer (PMDA: 2.22 g; NTCDA: 2.71 g) was added and stirred for 3 h at 0–5 °C. The pre-polymerization reaction was stopped once a brownish viscous solution was formed, and the mixture was transferred to a Teflon-inner autoclave for further polymerization at 180 °C for 10 h. After cooling to room temperature, the obtained product was rinsed with DMF and anhydrous ethanol, then two aromatic polyimides were obtained after vacuum drying at 40 °C for 24 h. The PI prepared by PMDA and BZD was named as PDPI and that obtained by NTCDA and BZD was denoted as NDPI. The preparation process of PI is depicted in Scheme 2.

### 2.4 Preparation of composite coatings

The composite coatings were prepared by ultrasonic dispersion of corrosion inhibitors (PDPI and NDPI) in different proportions CBCP, where neat CBCP was used as a control sample. In the first place, polyimide was dispersed into xylene by ultrasound, then added CBCP and continued to oscillate under ultrasound for about 30 seconds until complete dispersion. The as-obtained coatings were evenly coated on surfaces of the metal substrates. The corrosion inhibitor concentrations based on CBCP mass were set to 0.1 wt%, 0.5 wt%, and 1.0 wt%. The corresponding obtained coatings were denoted as (CP-01, CP-05, and CP-10) and (CN-01, CN-05, and CN-10) respectively.

### 2.5 Characterization

#### 2.5.1 Chemical structure

Fourier transform infrared spectroscopy (FTIR) were carried out on a Nicolet 5700 spectrometer in the range of 500–4000 cm^−1^. The ^1^H NMR spectra were recorded on a Bruker 400 MHz NMR spectrometer, using CDCl_3_ as the solvent. Solid-state ^13^C NMR experiments on a Bruker AVANCE NEO 400 NMR spectrometer operating. X-Ray photoelectron spectroscopy (XPS) was performed on a VG MultiLab 2000 XPS spectrometer (Thermo Electron, U.S.A.). All the binding energies were calibrated by C_1s_ peak at 284.8 eV.

#### 2.5.2 Evaluation of mechanical properties

The commercial tinplate as substrates for the mechanical properties testing. Before tests, the tinplate pieces were sanded to remove any rust and metal oxides present on the metal surface followed by, cleaning with alcohol and deionized water before air drying. Both the evaluation of mechanical properties was carried out at room temperature and about 50% relative humidity.

##### 2.5.2.1 Surface hardness test

The surface hardness of each coating was evaluated by pencil hardness testing according to standard GB/T6739-2006. Place the paint sample on a level, firm surface and use a pencil of different hardness levels to run across the surface of the coating until no scratches greater than 3 mm appear. 9H is the highest grade on the pencil hardness test and 9B is the lowest grade.

##### 2.5.2.2 Impact resistance test

The impact resistance was measured according to GB/T1732-2020 standard, where higher values meant, better fracture toughness. The test plate is a tinplate with size of 50 mm × 120 mm × 0.3 mm, the hammer is then sent from various heights through free fall to the surface of the test plate. The front and back of the test board shall be tested.

##### 2.5.2.3 Adhesion test

Two test methods were used to evaluate adhesion. The adhesion tests were performed according to the Chinese standards GB/T9286-2021 and GB/T1720-2020. Continuous squares or circles drew on the surface of the coating, and then determine the adhesion level by judging the peeling degree of the coating. The lower the grade, the better the adhesion.

##### 2.5.2.4 Flexibility test

The flexibility was tested according to the standard GB/T6742-2007. The back of the test plate is folded 180° around the cylinder of different diameters until the coating surface does not fall off after folding. The larger the diameter of the cylinder, the less flexible it is.

#### 2.5.3 Anti-corrosion tests

In order not to affect the result of the electrochemical test value, we used copper pieces with better conductivity were used as the substrates of the anti-corrosion experiment. Before coating, the copper pieces were sanded to remove any rust and metal oxides from the substrate. Afterward, the substrates were cleaned with deionized water and ethanol, and then air-dried. The anticorrosion performances were tested in a CHI660E electrochemical workstation (Shanghai Chenhua Instrument Co., Ltd, China) equipped with a common three-electrode cell. Data were recorded for the samples in 3.5 wt% NaCl and or 1.0 M HCl aqueous solution, respectively, at room temperature once the measured open circuit potential (OCP) stabilized. Platinum was used as a counter electrode, saturated calomel electrode as a reference, and coated substrates as working electrodes. The frequency range of the electrochemical impedance spectroscopy (EIS) measurements was from 10^5^ Hz to 10^−2^ Hz, and the amplitude perturbation was 5 mV. The scanning rate in Tafel polarization curves was 0.01 V s^−1^. The surface morphologies of corroded coating were observed by field emission scanning electron microscopy (SEM, SU8010).

#### 2.5.4 Thermogravimetric analysis

The thermogravimetric analysis (TG) of corroded coatings was performed by a TGA/SDTA851 thermogravimetric analyzer. The samples were heated from 30 °C to 600 °C under N_2_ atmosphere at the rate of 10 °C min^−1^.

## 3. Results and discussion

### 3.1 Synthesis and mechanical properties of CBCP

**Fig. 1:**
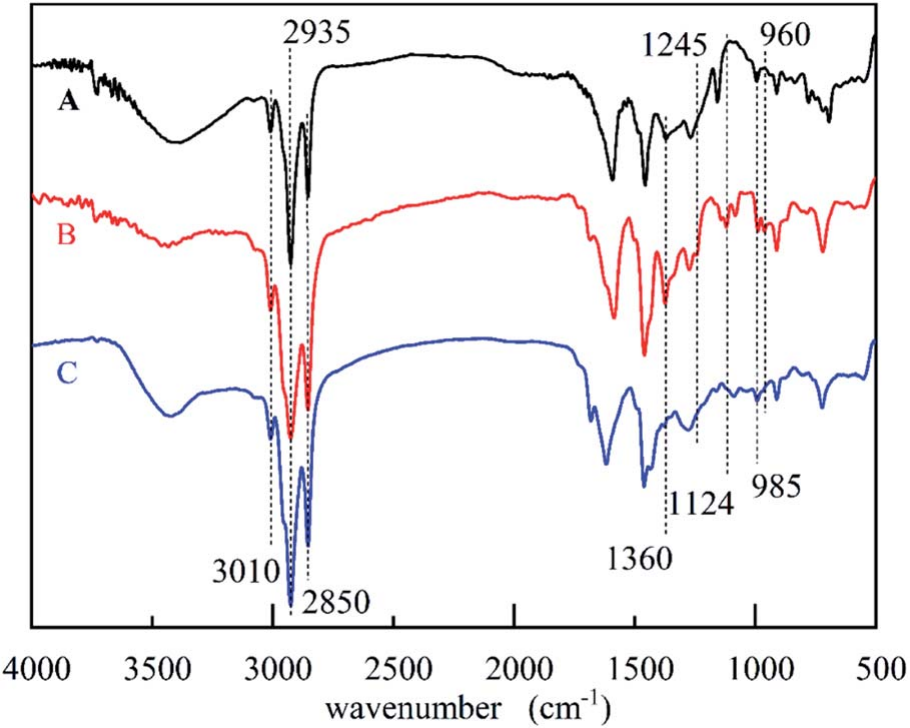
The FTIR spectra of cardanol (A), CB (B) and CBCP (C).

The solvent-free synthesis of CB could reduce the use of organic solvents. The FT-IR spectra of cardanol, CB, and CBCP were displayed in Fig. 1. The peaks at 2850 cm^−1^ and 2935 cm^−1^ were assigned to the stretching vibration of saturated C–C bonds on the side chain of cardanol. The peak at 3010 cm^−1^ was attributed to the stretching vibration of the isolated double bond. The characteristic peak of CB at 960 cm^−1^ was related to C–H out-of-plane bending vibration on benzoxazine ring, while the oscillation vibration of –CH_2_– was recorded at 1360 cm^−1^.^30^ The symmetric and asymmetric stretching vibration peaks of C–N–C on benzoxazine ring appeared at 1035 cm^−1^ and 1245 cm^−1^. The peak at 1124 cm^−1^ was attributed to the asymmetric stretching vibration of C–N–C. All these the results were consistent with previous reports, indicating the successful synthesis of CB.

**Fig. 2:**
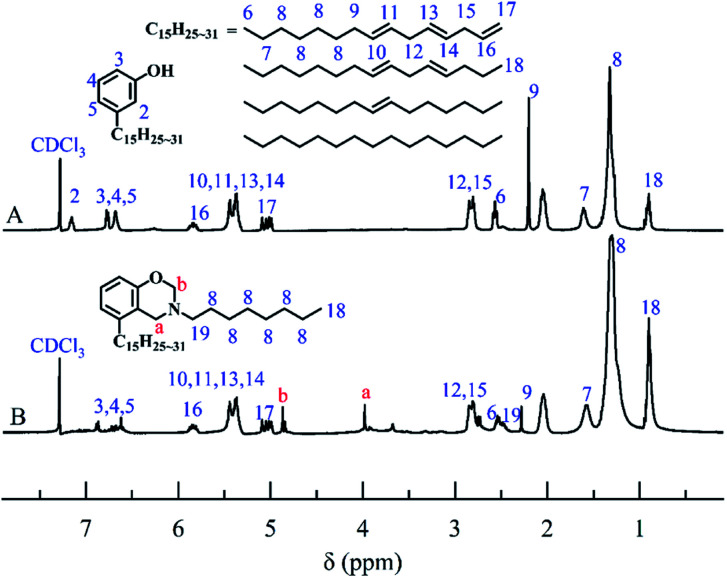
The ^1^H-NMR spectra of cardanol (A) and CB (B).

To further confirm the structure of CB, ^1^H-NMR analyses of cardanol and CB were performed. As shown in Fig. 2, the peak ranging from 6.60 to 7.10 ppm was attributed to hydrogen atoms present on benzene ring of cardanol, while the solvent peak of CDCl_3_ was recorded at 7.28 ppm. The displacement peaks at 0.89–2.84 ppm and 4.89–5.87 ppm were attributed to the saturated fat chain and hydrogen atoms on the unsaturated double bond of cardanol, respectively. Obviously, singlet state proton characteristic peaks appeared at 4.86 ppm and 3.97 ppm, corresponding to O–CH_2_–N and Ar–CH_2_–N in benzoxazine ring, respectively.^31,32^ These results further confirmed the successful synthesis of CB.

Heat curing was commonly used in benzoxazine. Where ring-opening polymerization may occur at high temperatures above 180 °C. However, such high reaction temperatures were not suitable for practical applications. In this paper, the CuCl_2_ catalyzed ring-opening polymerization method was used to prepare CBCP. This route significantly reduced the ring-opening temperature. The FT-IR spectra of CB and CBCP in Fig. 1 showed vanishing characteristic peaks of benzoxazine at 960 cm^−1^ and 1360 cm^−1^ after CuCl_2_ catalytic polymerization. The symmetric and asymmetric stretching vibration peaks of C–N–C at 1035 cm^−1^ and 1245 cm^−1^, as well as the asymmetric stretching vibration peaks of C–N–C at 1124 cm^−1^ also disappeared.^33^ Thus, the CB monomer underwent ring-opening polymerization, and CuCl_2_ successfully catalyzed the ring-opening reaction of CB.

**Fig. 3:**
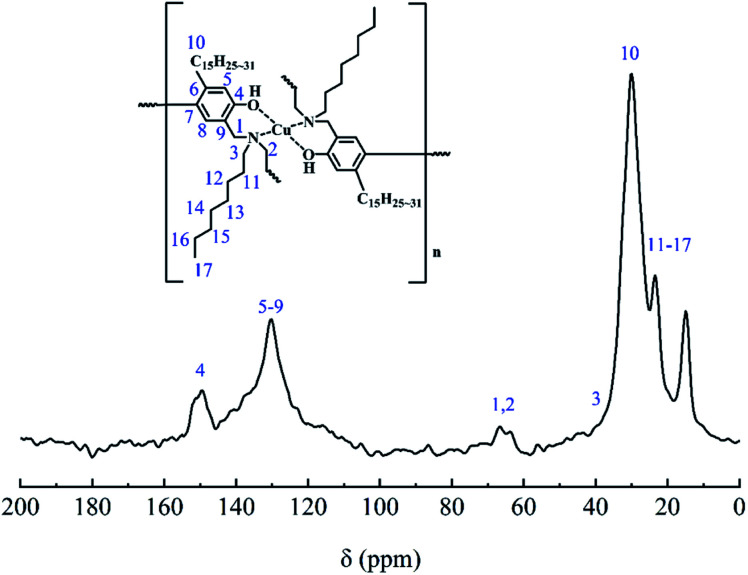
Solid-state ^13^C NMR spectra of CBCP.

Fig. 3 shows the solid-state CP/MAS ^13^C-NMR spectra of CBCP. As shown in Fig. 3, the peaks at 66.44 and 63.81 ppm correspond to the resonances of methylene carbons (C_1_ and C_2_) of O–CH_2_–N and Ar–CH_2_–N, respectively.^34^ Some chemical shifts (ppm) were assigned as follows: 40.13 (C_3_), 149.76 (C_4_), 30.19 (C_10_), and the other resonances of C_11_, C_12_, C_13_, C_14_, C_15_, C_16_, C_17_ and carbon on the side chain of cardanol were highly overlapped in the chemical shift rang of 7.97–38.08 ppm.^35,36^ Other chemical shifts (ppm) were the aromatic carbon resonances: C_5_, C_6_, C_7_, C_8_, and C_9_ were highly overlapped in the chemical shift rang of 111.46–143.04 ppm.^37^ In addition, X-ray photoelectron spectroscopy (XPS) analysis was performed on the surfaces of CBCP cured by heating at 200 °C for 2 h and CBCP catalyzed by CuCl_2_, see Fig. S2(a–h).† These chemical shifts of the carbons confirm the CBCP was successfully prepared.

Since the crosslinking degrees of polymers vary with heating temperature, the physical and mechanical properties of polymer coatings may also vary with temperature. In this work, CBCP was heated at different temperatures for 2 h, and its physical and mechanical properties were tested accordingly (Table 1). Eqn (1) was used to calculate the gelling rate:

1 The gel rate = (*m*_1_/*m*_2_) × 100%

where *m*_1_ is the initial mass before soaking and *m*_2_ refers to the remaining solid mass of CBCP after soaking in xylene for 2 h.^38^ As shown in Table 1, the gelling rate of CBCP increased with temperature. At 120 °C, the gelling rate was estimated to about 100%. Therefore, CBCP could be polymerized at 120 °C under CuCl_2_ catalysis. As the heating temperature increase, the gelling rate and hardness of CBCP increased. However, the impact strength, adhesion, and flexibility decreased. At a heating temperature of 100 °C, the bending tests withstood only 10 mm diameter samples. Similarly, CBCP coating heated at 40 °C and 60 °C became completely dissolved during gelling rate tests. As a result, no cross-linking occurred in the polymer under short-time low-temperature heating. Hence, the material would more likely to exist in the form of coordination polymerization.

**Table 1:** Performances of CBCP at different heating temperatures

Properties		a	b	c	d	e	F	g	h	i
		25 °C^a^	40 °C	60 °C	80 °C	100 °C	120 °C	140 °C	160 °C	180 °C
Pencil hardness		2H	B	B	2H	4H	6H	6H	6H	6H
Impact strength (g cm^−1^)	Obverse	50 000	50 000	35 000	25 000	20 000	15 000	15 000	15 000	15 000
	Reverse	50 000	35 000	15 000	15 000	15 000	15 000	15 000	15 000	15 000
Adhesive force (grade)	Circle	1	1	1	1	2	2	2	3	3
	Cross	0	0	0	0	1	1	2	3	3
Flexibility (mm)		2	2	2	2	10	10	10	10	10
The gel rate (%)		73	0.5 h^b^	0.5 h^b^	62	92	100	100	100	100

a Coatings not heated and placed for two weeks before testing.

b Time for coatings to completely dissolve in xylene.

Therefore, CBCP was tested after dried at room temperature two weeks and the results showed gelling rate reaching 73% and pencil hardness is 2H. Also, no discoloration of solvent or coating dissolution took place. The results also revealed excellent impact strength, adhesion, and flexibility of this material dried naturally. By comparison, though the hardness and gel rate of CBCP can be improved by increasing the heating temperature, features like impact strength, adhesion and flexibility may constantly reduce. Here, naturally dried CBCP coating at room temperature underwent orderly and slow cross-linking polymerization, enabling better balanced internal structure to cope with the damage caused by external forces.

### 3.2 Synthesis of PDPI and NDPI

**Fig. 4:**
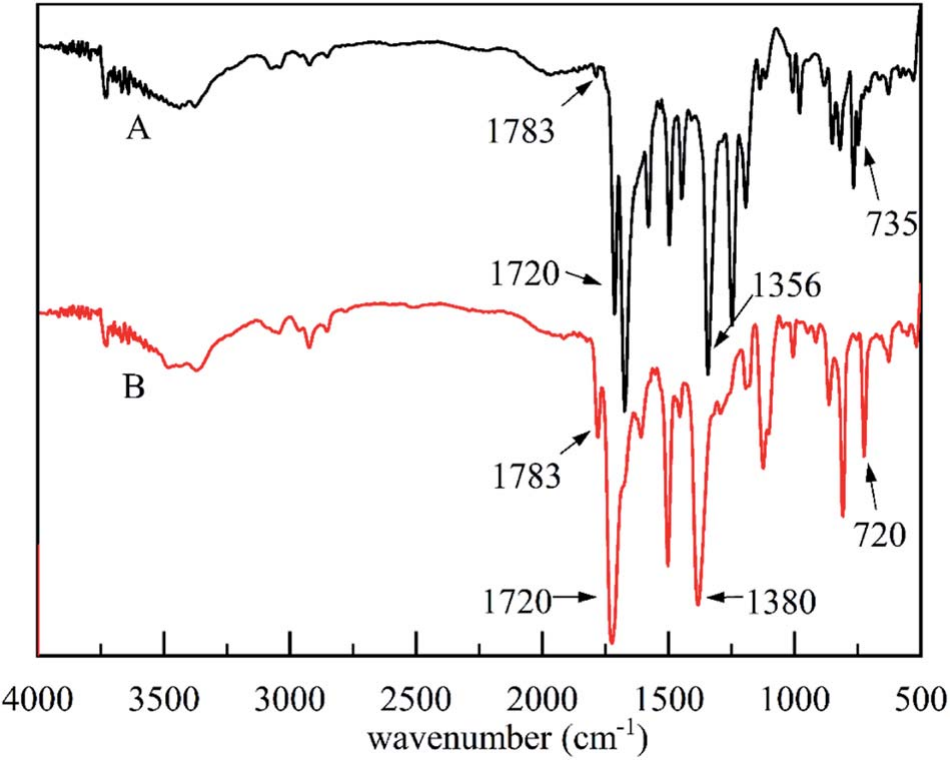
The FT-IR spectra of PDPI (A) and NDPI (B).

The FT-IR spectra of PDPI (A) and NDPI (B) were provided in Fig. 4. The stretching vibration characteristic peaks of asymmetric carbonyl groups in polyimide were observed at 1783 cm^−1^ both PDPI and NDPI. The out-of-plane bending vibration characteristic peaks appeared at 735 cm^−1^ and 720 cm^−1^, respectively. The peak at 1720 cm^−1^ was assigned to the symmetrical carbonyl stretching vibration. The amide bond as an important characteristic absorption peak of aromatic polyimide appeared at 1356 cm^−1^. These results agreed well with previous reports.^39^

**Fig. 5:**
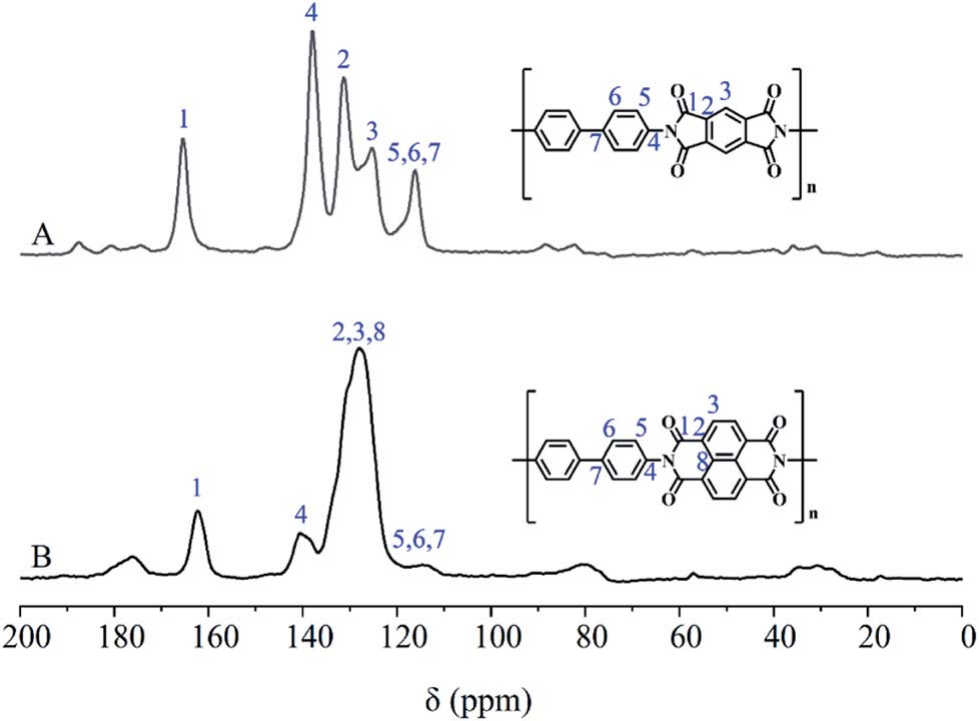
Solid-state ^13^C NMR spectra of (A) PDPI and (B) NDPI.

The structure of PDPI and NDPI was further characterized by solid-state ^13^C CP/MAS NMR, as shown in Fig. 5. The peaks at 165.42 (A) and 162.33 (B) ppm correspond to the resonances of carbonyl carbons on an amide (C_1_) of PDPI (A) and NDPI (B) respectively.^40^ Other chemical shifts (ppm) were the aromatic carbon resonances: C_2_, C_3_, C_4_ C_5_ C_6_, C_7_, and due to the aromatic carbons on benzidine (C_5_, C_6_, C_7_) have similar chemical environments were highly overlapped in the chemical shift rang of 110.35–121.81 ppm.^41^ In addition, on account of NDPI has more aromatic carbons (C_2_, C_3_, C_8_) than PDPI, the integral area ratios of C_2_ and C_3_ in NDPI were significantly higher and overlapped.^42^ All the results indicating the successful preparation of PDPI and NDPI. The surface morphology of corrosion inhibitors in Fig. S1† revealed the two-dimensional sheet structures of PDPI and NDPI and the mobility test for water and solvent resistance of PDPI and NDPI in Fig. S3.†

### 3.3 Electrochemical properties of composite coatings

A three-electrode system (working electrode, counter electrode, and reference electrode) was used to study the corrosion resistance characteristics of the coatings CBCP (O), CP-01 (A), CP-05 (B), CP-10 (C), CN-01 (D), CN-05 (E), and CN-10 (F). Solutions containing 3.5 wt% NaCl and 1 M HCl were employed as corrosion media. The coatings were first immersed in the corrosive medium at open circuit potential (OCP) to ensure steady-state conditions. The corrosion current (*I*_corr_) and corrosion voltage (*E*) of each coating were then obtained by exploring the polarization Tafel curves.^43–45^ The polarization resistance (*R*_p_), protection efficiency (PE) and corrosion rate (CR) were calculated by (eqn (2)–(4), respectively):2
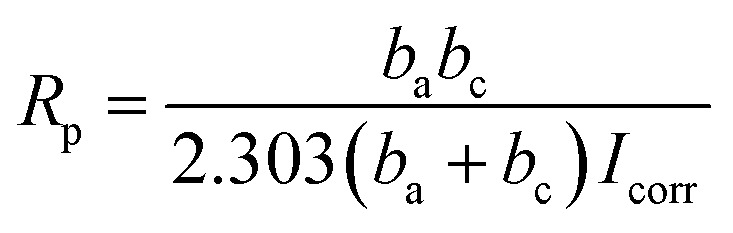


In Tafel curves, the current corresponding to the intersection of the slope of anode and cathode curves represented the corrosion current (*I*_corr_). The slopes of both anode and cathode curves *b*_a_ and *b*_c_ were obtained by fitting analysis of the testing software.3
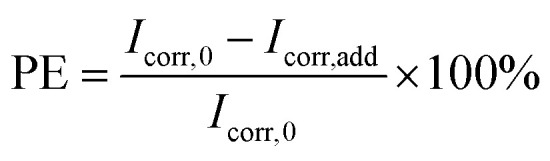
where, *I*_corr,0_ represents the corrosion current of the control sample, and *I*_corr,add_ refers to the corrosion current of the experimental sample.4
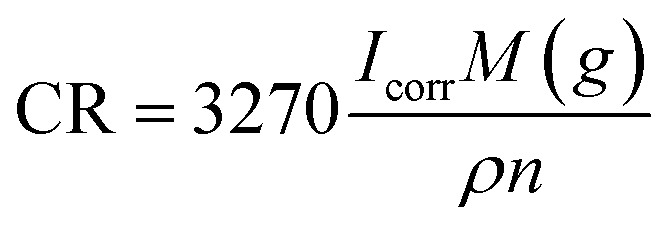
where *A* is the atomic mass of the metal (*A* = 63.5), *ρ* refers to the density of the metal (*ρ* = 8.5 g cm^−3^), and *n* denotes the valence state of the metal ion (*n* = 2).

**Fig. 6:**
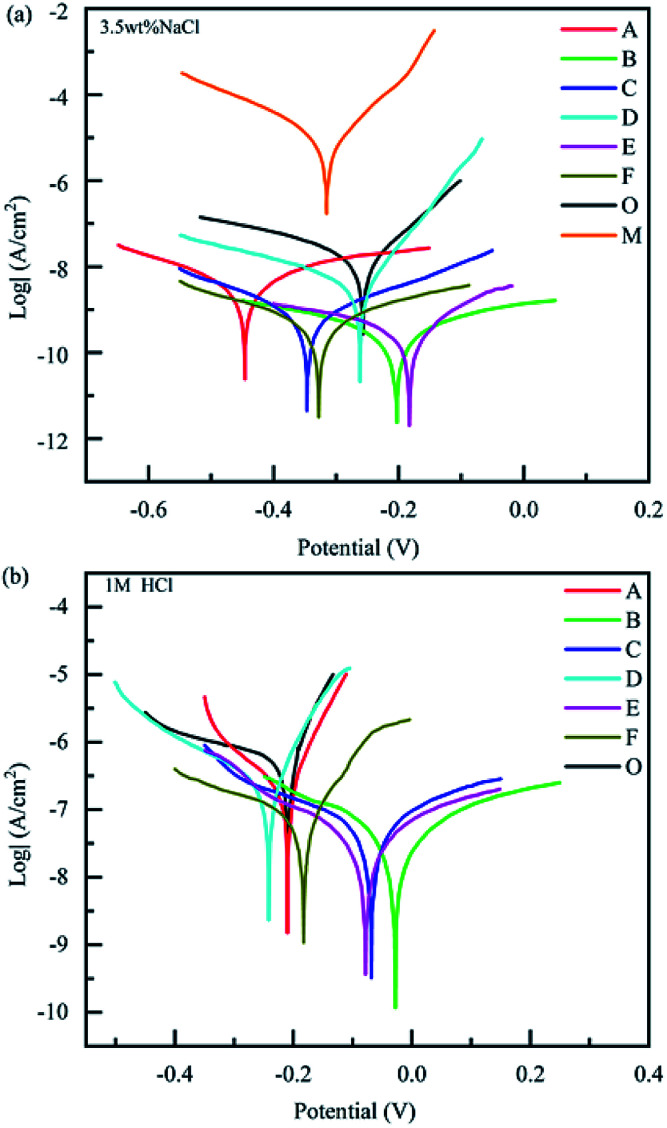
Tafel curves of different coatings in (a) 3.5 wt% NaCl solution and (b) 1 M HCl aqueous. Bare copper (M), CBCP (O), CP-01 (A), CP-05 (B), CP-10 (C), CN-01 (D), CN-05 (E), and CN-10 (F).

In general, the corrosion degree of each coating can preliminarily be evaluated though *I*_corr_ and *E* in Tafel curves.^25^ The coatings with lower *I*_corr_ and higher *E* exhibited better corrosion resistance. In 3.5 wt% NaCl solution, the corrosion resistance of bare copper was poor. By comparison, the samples protected by coatings all showed excellent corrosion resistance (Fig. 6(a)). As shown in Table 2, the different values of *R*_p_, PE, and CR, indicating variable corrosion resistances. Compared to bare copper, the *I*_corr_ of CBCP coating decreased to 1.369 × 10^−8^ A cm^−2^ and *E* increased to −0.2585 V. The *R*_p_ of CBCP was 1.120 × 10^8^ Ω, CR was 1.672 × 10^−4^ mm year^−1^, and PE of metal was 99.82%. Hence, CBCP possessed good corrosion resistance performance when coated on the metal substrate.

**Table 2:** Relevant chemical parameters extracted from Tafel curves in 3.5 wt% NaCl solution

Samples	*E* (V)	*I*_corr_ (A cm^−2^)	*R*_p_ (Ω)	PE (%)		CR (mm year^−1^)
Bare copper	−0.3160	7.914 × 10^−6^	2.487 × 10^5^	0^a^	—a	9.666 × 10^−2^
CBCP	−0.2585	1.369 × 10^−8^	1.120 × 10^8^	99.82	0	1.672 × 10^−4^
CP-01	−0.4460	3.659 × 10^−9^	3.035 × 10^8^	99.96	73.28	4.469 × 10^−5^
CP-05	−0.2042	2.764 × 10^−10^	3.874 × 10^9^	99.99	97.98	3.376 × 10^−6^
CP-10	−0.3470	7.580 × 10^−10^	1.720 × 10^9^	99.99	94.46	9.259 × 10^−6^
CN-01	−0.2620	3.083 × 10^−9^	5.414 × 10^8^	99.96	77.48	3.765 × 10^−5^
CN-05	−0.1830	3.373 × 10^−10^	3.385 × 10^9^	99.99	97.54	4.120 × 10^−6^
CN-10	−0.3280	5.112 × 10^−10^	2.183 × 10^9^	99.99	96.26	6.244 × 10^−6^

a 0 – based on this sample; — – excluded this sample.

Proper amount of corrosion inhibitor could enhance the corrosion resistance of coatings. Here, the *I*_corr_ and CR of composite coatings were lower than those of CBCP coatings, and protection efficiency greater than 70% when compared to CBCP (Table 2). Both CP-05 and CN-05 showed excellent corrosion resistance, with *I*_corr_ to 2.764 × 10^−10^ A cm^−2^ and 3.373 × 10^−10^ A cm^−2^, CR calculated as 3.376 × 10^−6^ mm year^−1^ and 4.120 × 10^−6^ mm year^−1^ and *R*_p_ valued to 3.874 × 10^9^ Ω and 3.385 × 10^9^ Ω, respectively. Compared to CBCP, the protection efficiency (PE) of CP-05 and CN-05 reached values of 97.98% and 97.54%, respectively. Also, PE reached 99.9% when compared to bare copper. Overall, the corrosion resistance of CBCP coating significantly improved after the introduction of polyimide.

The Tafel curves of composite coatings were also investigated in different media, and then, 1 M HCl solution as the corrosive medium. Fig. 6(b) and Table 3 showed the Tafel curves and parameters of the coatings in 1 M HCl solution, respectively. The anti-corrosion performance of the coatings in acidic media was slightly worse than in neutral media. However, the anti-corrosion effects of the composite coatings were still remarkable. Compared to CBCP, the PE values of CP-05 (B) and CN-05 (E) reached 94.87% and 93.24%, respectively. Both CP-10 (C) and CN-10 (F) showed similar performances in acidic media as in neutral media. Also, their corrosion resistances decreased slightly when compared to CP-05 (B) and CN-05 (E). Therefore, excess amounts of polyimide inhibited the anticorrosion effect of the coatings. When polyimide was not evenly dispersed in CBCP, the compactness of the coatings became compromised, thereby weakening the protective ability of the coatings on metal materials.

**Table 3:** Relevant chemical parameters extracted from Tafel curves in 1 M HCl solution

Sample	*E* (V)	*I*_corr_ (A cm^−2^)	*R*_p_ (Ω)	PE (%)	CR (mm year^−1^)
CBCP	−0.210	6.009 × 10^−7^	1.347 × 10^6^	0^a^	7.340 × 10^−3^
CP-01	−0.210	2.447 × 10^−7^	1.407 × 10^6^	59.28	2.989 × 10^−3^
CP-05	−0.028	3.080 × 10^−8^	4.121 × 10^7^	94.87	3.762 × 10^−4^
CP-10	−0.068	8.146 × 10^−8^	1.049 × 10^7^	86.44	9.945 × 10^−4^
CN-01	−0.229	2.511 × 10^−7^	5.275 × 10^6^	58.21	3.067 × 10^−3^
CN-05	−0.078	4.601 × 10^−8^	2.556 × 10^7^	93.24	5.620 × 10^−4^
CN-10	−0.183	8.724 × 10^−8^	1.180 × 10^7^	81.23	1.066 × 10^−3^

a 0 – based on this sample.

Some of the best anti-corrosion coatings reported in literature were prepared with cardanol-based composite coatings, and the PE values of the two-dimensional lamellar polyimide/cardanol-based benzoxazine copper polymer composite coatings prepared here were compared with other results in literature in Table 4. Among previously reported coatings, the best PE value of 99.87% was reported for a 5 wt% HTCP/SBCBz-EP/MS composite coating (SBCBz-EP: siloxane-based cardanol benzoxazine–epoxy). Notably, the PE value of the two-dimensional lamellar polyimide/cardanol-based benzoxazine copper polymer composite coating (CP-05) in this work increased by 99.99% compared with bare copper and 97.98% compared with the pure matrix (CBCP), both of which were significantly better than previous coatings reported in literature. In conclusion, the combination of polyimide and cardanol-based benzoxazine copper polymer significantly improves the protection efficiency of the coating.

**Table 4:** Protection efficiency of anti-corrosion coatings reported in literature

Coatings	Year	PE (%)	Ref.
CF PBz/bio-silica coated specimen-7	2020	75.00	46
Self-healing coating-16%	2022	86.92	47
5 wt% f-CDC/HSBBz-EP/MS	2021	99.40	48
5 wt% HTCP/SBCBz-EP/MS	2020	99.87	49
HEFPI	2016	99.00	50
Cardanol conc. (ppm)-200	2019	89.30	51
This work		99.99	

**Fig. 7:**
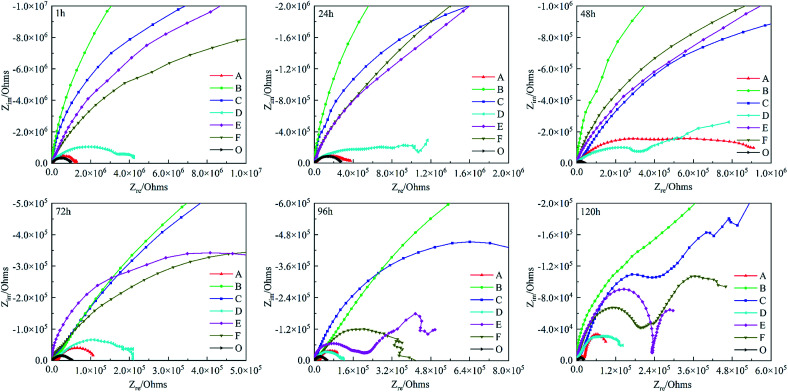
Nyquist plots of different coatings in 3.5 wt% NaCl solution. CBCP (O), CP-01 (A), CP-05 (B), CP-10 (C), CN-01 (D), CN-05 (E), and CN-10 (F).

To study the influence of polyimide corrosion inhibitors, the corrosion resistance of different coatings CBCP (O), CP-01 (A), CP-05 (B), CP-10 (C), CN-01 (D), CN-05 (E) and CN-10 (F) were evaluated by EIS in 3.5 wt% NaCl solution for 1–120 h. In Fig. 7, the capacitance arcs in the low/high-frequency region were related to the transfer and charge transfer of corrosion reaction.^25^ Therefore, the corrosion resistance of the coatings can be judged by the diameters of the curves. The local EIS spectrograms revealed all coatings with large capacitive arcs at the initial stage of immersion. The capacitance arcs of CP-05 (B), CP-10 (C), CN-05 (E), and CN-10 (F) looked relatively large, indicating better corrosion resistance. By comparison, the capacitance arcs of CBCP were the smallest, consistent with the results of Tafel polarization. As testing time increased, the diameters of all capacitive arcs decreased. The reason for this had to do with the continuous erosion of the coatings by the corrosive medium, which resulted in holes and cracks, as well as partial rupture of the protective layer. At the test times of 96 h and 120 h, the materials CP-10 (C), CN-05 (E) and CN-10 (F) showed multiple capacitive arcs with small radii in the high-frequency region. Thus, the corrosive medium penetrated the coatings through some aperture, but did not yet reach the surface of the metal, keeping it protected.^52^ CP-05 (B) still showed a large capacitive arc resistance, revealing excellent corrosion resistance. Polyimide compound benzoxazine resin as a protective layer of metal materials could effectively delay the infiltration of water and other corrosive media, thereby preventing the metal from corrosion.

**Fig. 8:**
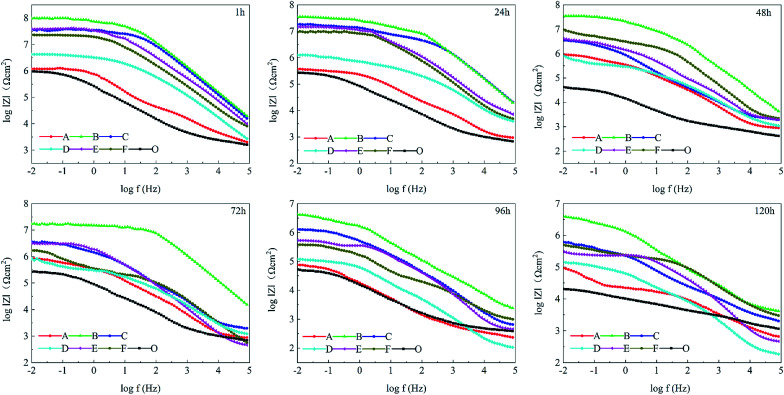
Bode plots of different coatings in 3.5 wt% NaCl solution. PBzn (O), P1-01 (A), P1-05 (B), P1-10 (C), P2-01 (D), P2-05 (E), and P2-10 (F).

**Fig. 9:**
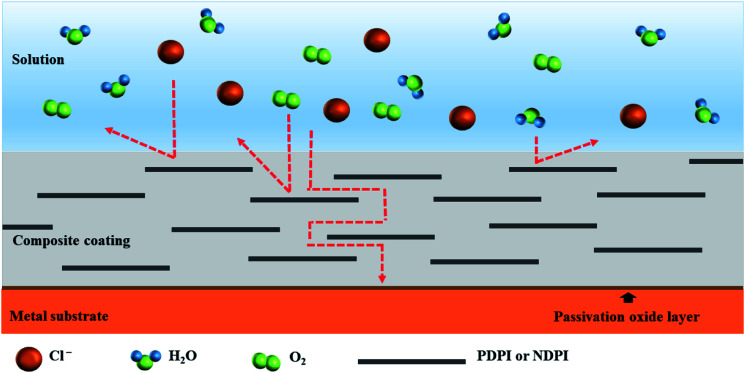
Schematic diagram of anticorrosion mechanism of composite coatings.

The changes in EIS Bode plots of the coatings immersed in 3.5 wt% NaCl solution were illustrated in Fig. 8. The Bode plots revealed CP-05 (B) with only one time constant in the late test period. It also maintained a high impedance modulus over a wide frequency range. Low-frequency impedance modulus (|*Z*|_f_ = 0.01 Hz) was often used to evaluate the corrosion resistance of coatings.^53^ CP-05 (B) showed a high impedance modulus in the low-frequency region of Bode plots, indicating the good corrosion resistance of the coating. The composite coatings showed good anticorrosion properties, mainly in the following aspects (as shown in Fig. 9): firstly, the higher cross-linking density cardanol-based benzoxazine film was responsible for the good shielding effect. Secondly, though corrosive mediums are known to easily penetrate coatings through tiny surface cracks, two-dimensional lamellar polyimide can effectively avoid and prevent it from accessing the metal surface. PDPI or NDPI that played an important role in the maze effect and reduction the corrosion rate of the copper pieces. Thirdly, the nitrogen atom of organic amine compounds usually has lone pairs of electrons, which can form coordination bonds with the hollow d orbitals of the metal, such that the molecule adsorbed on the metal surface forms a layer of protective film to protect the metal.^54^

**Fig. 10:**
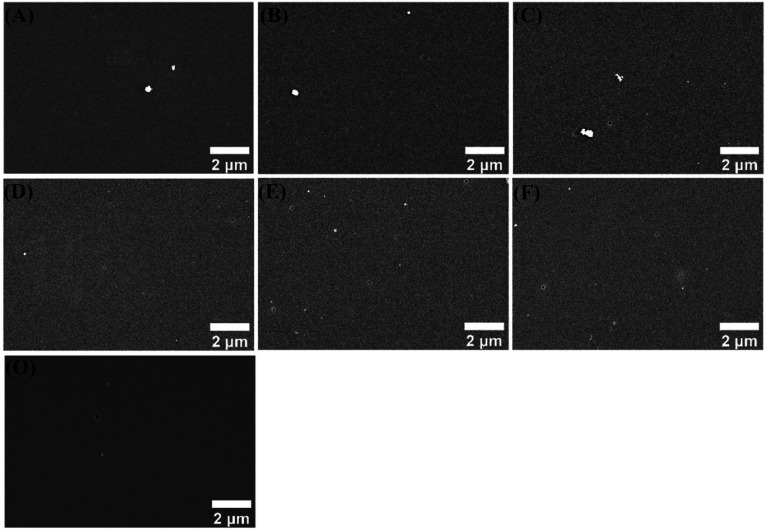
SEM images of different coatings immersed in 3.5 wt% NaCl solution for 120 h. CP-01 (A), CP-05 (B), CP-10 (C), CN-01 (D), CN-05 (E), CN-10 (F), and CBCP (O).

The surface morphologies of different coatings in 3.5 wt% NaCl solution for 120 h were gathered in Fig. 10. The surface of CBCP coating looked smoother and flatter, while the composite coatings revealed relatively rough surfaces. This might be related to the dispersion of polyimide in CBCP. Serious corrosion pits and holes appeared on CBCP surface after corrosion since incomplete cross-linking of CBCP may result in rapid penetration of the coatings by the corrosive media. The composite coatings did not exhibit large holes, but many small corrosion pits and holes were noticed. Since the added amounts of the corrosion inhibitors in CP-01 (A) and CN-01 (D) were small, they were incapable of completely shielding the corrosive media. On the other hand, large numbers of corrosion pits existed on coatings surfaces though CP-10 (C) and CN-10 (F) showed some small holes after 120 h immersion in 3.5 wt% NaCl solution, indicated the presence of corrosion inhibitors enhanced corrosion resistance. The corrosion resistance of CP-10 (C) and CN-10 (F) may be caused by excess added amounts, resulting in uneven dispersion of corrosion inhibitors in the solvent.^55^ CP-05 (B) showed excellent anticorrosion performances in all composite coatings with no obvious corrosion holes and only some corrosion pits. Therefore, CP-05 (B) exerted a good shielding effect on the metal in corrosive media when compared to coatings, showing excellent anti-corrosion performance.

### 3.4 Thermogravimetric analysis

**Fig. 11:**
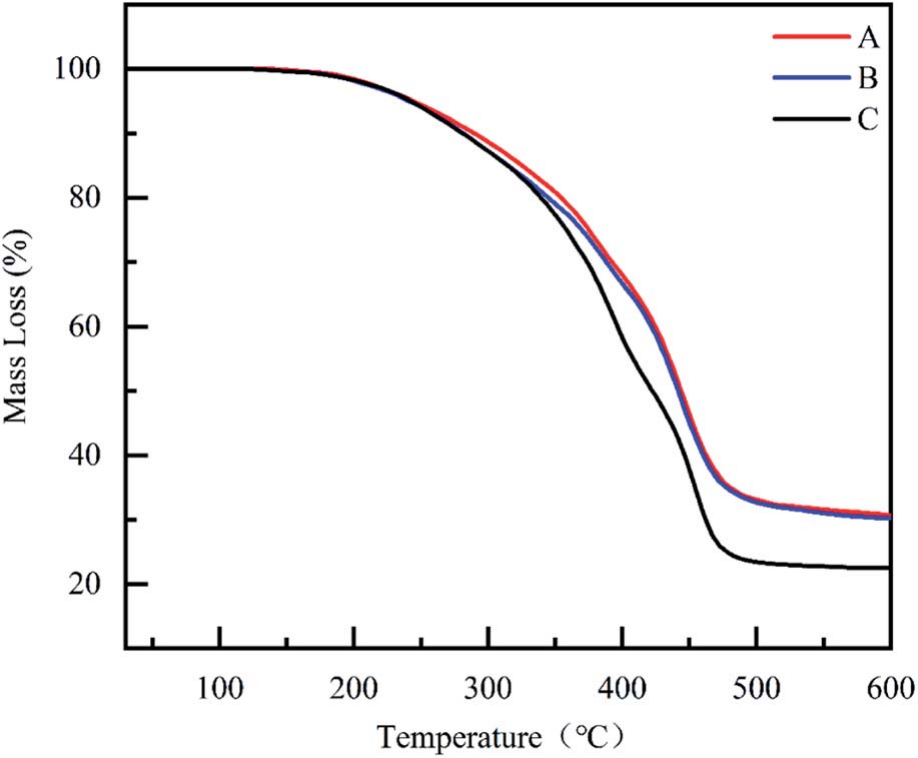
Thermogravimetric analysis of, CN-05 (A), CP-05 (B) and CBCP (C).

To clarify the thermal properties of the composite coatings, TGA curves were obtained. TGA provides important information about the degradation behavior of the material and thus explains the thermal stability of the material.^56–58^ As shown in Fig. 11, all coatings exhibited good stability below 200 °C. Compared to CBCP coating, the composite coatings CP-05 and CN-05 illustrated better thermal stabilities. The heat resistance index temperature (THRI) was calculated using eqn (5) and it was presented in Table 5.^59,60^

5 THRI = 0.49 × [T5 + 0.6 × (T30 − T5)]

**Table 5:** Extracted thermogravimetric analysis data of CN-05, CP-05, and CBCP

Samples	5% weight loss [T5] (°C)	30% weight loss [T30] (°C)	THRI (°C)	Char yield at 600 °C (%)
CN-05	245.3	392.6	163.5	30.1
CP-05	240.6	387.3	161.0	29.2
CBCP	240.4	372.1	156.5	22.4

The data relating to 5% weight loss temperature, 30% weight loss temperature, and carbon residue rate at 600 °C of all three coatings were listed in Table 5. The temperature corresponding to a weight loss rate of 5% can be considered as the initial decomposition temperature of the coatings and the low-molecular weight oligomers were volatilized.^61^ The difference in thermal stability of the coatings was most obvious at a weight loss rate of around 50%. As temperature rose to 600 °C, the carbon residue rates of CBCP, CP-05, and CN-05 reached 22.4%, 29.2% and 30.1%, respectively. The reason for this result might be the small anti-corrosion inhibitor added amounts that led to no obvious differences in heat resistance between the composite coatings. Therefore, the addition of PDPI and NDPI effectively improved the thermal stability and carbon residue rate of CBCP. The high decomposition temperature of composite coatings would greatly help practical applications.

## 4. Conclusions

CB was successfully synthesized from cardanol, *n*-octylamine, and paraformaldehyde by the solvent-free method. CuCl_2_ was used as a catalyst of CB ring-opening reaction to from CBCP and could be cured at room temperature and exhibited mechanical properties. Meanwhile, two aromatic polyimides (PDPI and NDPI) were synthesized and added to CBCP coating as corrosion inhibitors. All composite coatings showed better anticorrosion performances than CBCP coating. Especially, the composite coating (CP-05) obtained with 0.5 wt% PDPI illustrated the best anti-corrosion performance. The addition of aromatic polyimide also improved the heat resistance of the composite coatings. As a result, the designed two-dimensional lamellar polyimide/cardanol-based benzoxazine copper polymer composite coatings possessed good mechanical properties and excellent anti-corrosion performances. In sum, it has broad application prospect for future metal anticorrosion application that the suggested facile and eco-friendly strategy for preparing bio-based anticorrosive composite coatings from low cost and abundant cardanol and polyimide corrosion inhibitors.

## Author contributions

Xiangyang Chen: investigation, methodology, data curation, writing–original draft, writing–review & editing. Xinmei Zhang: investigation, validation. Jipeng Chen: investigation, validation. Weibin Bai: conceptualization, methodology, writing–review & editing. Xiaoxiao Zheng: conceptualization, methodology, writing–review & editing. Qi Lin: conceptualization, funding acquisition. Fengcai Lin: conceptualization, methodology, writing–review & editing. Yanlian Xu: conceptualization, methodology, writing–review & editing, supervision, project administration, funding acquisition.

## Conflicts of interest

The authors declare that they have no known competing financial interests or personal relationships that could have appeared to influence the work reported in this paper.

## Supplementary Material
